# A New Cadinane Sesquiterpene from the Marine Brown Alga *Dictyopteris divaricata*

**DOI:** 10.3390/molecules14062273

**Published:** 2009-06-24

**Authors:** Wei Wen, Fang Li, Nai-Yun Ji, Xiao-Ming Li, Chuan-Ming Cui, Xiao-Dong Li, Li-Na Zhang, Qin-Zhao Xue, Bin-Gui Wang

**Affiliations:** 1 Yantai Institute of Coastal Zone Research for Sustainable Development, Chinese Academy of Sciences, Yantai 264003, China; E-mails: wenwei_500@sina.com (W.W.), Imnli@163.com (X.-D.L.), Zhang.ln@163.com (L.-N.Z.), qzxue@yic.ac.cn (Q.-Z.X.); 2 Qingdao University of Science & Technology, Qingdao 266042, China; E-mail: iceli_2003@126.com (F.L.); 3 Institute of Oceanology, Chinese Academy of Sciences, Qingdao 266071, China; E-mails: lixmqd@yahoo.com.cn (X.-M.L.), chuanming-cui@163.com (C.-M.C.), wangbg@ms.qdio.ac.cn (B.-G.W.)

**Keywords:** *Dictyopteris divaricata*, sesquiterpene, cadinane

## Abstract

A sample of the marine brown alga *D. divaricata* collected off the coast of Yantai (P.R. China) was dried, powdered, and extracted with the mixture of CHCl_3_ and MeOH (1:1, v/v). By a combination of silica gel and Sephadex LH-20 column chromatography and preparative TLC, a new cadinane sesquiterpene 1,4-epoxymuurolan-5*β*-ol (**1**) was isolated from this species. Its structure was established by detailed MS and NMR spectroscopic analysis, as well as comparison with literature data.

## Introduction

**Figure 1:**
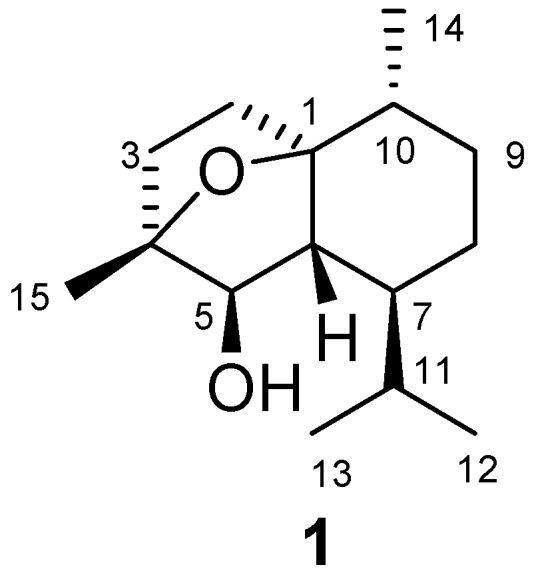
Structure of compound **1**.

Marine brown algae of the genus *Dictyopteris* are prolific sources of sesquiterpenes, and cadinane is a main carbon skeleton type [1,2,3,4,5,6,7,8]. In our investigations on the structurally interesting and biologically active terpenes from Chinese marine algae, we examined the chemical constituents of *D. divaricata* collected off the coast of Yantai and as a result, a new cadinane sesquiterpene, 1,4-epoxymuurolan-5*β*-ol (**1**) has been isolated and characterized for the first time. This paper reports the isolation and structural elucidation of compound **1** (Figure 1).

## Results and Discussion

The dried and powdered alga *D. divaricata* was extracted with a mixture of CHCl_3_ and MeOH (1:1, v/v). The concentrated extracts were partitioned between H_2_O and EtOAc. The EtOAc-soluble fraction was further purified by a combination of silica gel and Sephadex LH-20 column chromatography, as well as preparative TLC, to yield compound **1**. Compound **1** was obtained as a colorless oil. The broad IR absorption at *v*_max_ 3,452 cm^-1^ indicated the presence of a hydroxyl group in the molecule. The positive electrospray ionization mass spectrometry (ESIMS) exhibited a characteristic quasimolecular ion peak at *m*/*z* 261 [M+Na]^+^. The molecular formula was determined as C_15_H_26_O_2_ on the basis of HRESIMS (*m*/*z* 261.1829 [M+Na]^+^, calcd. for C_15_H_26_O_2_Na, 261.1830), suggesting three degrees of unsaturation. The ^1^H-NMR spectrum of **1** (Table 1) displayed one methyl singlet, three methyl doublets, and one broad singlet, attributed to an oxygenated methine. The ^13^C-NMR spectrum (Table 1), along with the DEPT and HSQC experiments revealed the presence of four methyl, four methylene, five methine, and two quaternary carbon atoms. A detailed comparison of the above spectra data with those reported for 1,4-epoxymuurolan-5*α*-ol revealed that **1** differed from this last compound mainly at C-5 (*δ*_C_ 85.5 d) [9], suggesting that compound **1** may be a C-5 isomer of 1,4-epoxymuurolan-5*α*-ol. The ^1^H-^1^H COSY correlations as shown in Table 1 and the observed HMBC correlations from H-12 to C-7, C-11, and C-13, from H-13 to C-7, C-11, and C-12, from H-14 to C-1, C-9, and C-10, from H-15 to C-3, C-4, and C-5, and from H-5 to C-3, C-4, C-6, C-7, and C-15 confirmed the planar structure of **1.** The relative configuration of **1** was determined by analysis of NOESY spectrum and coupling constants. The NOESY correlations between H-5 and H-7, H-2a indicated H-5, H-7 and C-2 to be located on the same face of the molecule. The same orientation of C-14 and C-2 was suggested on the basis of the NOESY correlation between H-14 and H-2b. H-6 and C-15 were assigned on the same face according to the observed NOESY correlation between H-6 and H-15. H-6 was located on the opposite face of H-7 based on the large coupling constant (11.6 Hz) between them. The above evidence established the structure of **1** to be 1,4-epoxymuurolan-5*β*-ol (Figure 1), the C-5 epimer of 1,4-epoxymuurolan-5*α*-ol [9]. Compound **1** was tested for the toxicity against brine shrimp (*Artemia salina*) [10]. However, it exhibited no toxicity against brine shrimp at 100 μg/mL.

## Experimental

### General

NMR spectra were recorded in CDCl_3_ with TMS as internal standard on a Bruker Avance 500 MHz NMR spectrometer operating at 500 and 125 MHz for ^1^H and ^13^C, respectively. Low and high resolution mass spectra were determined on a VG Autospec 3000 mass spectrometer. The IR spectrum was obtained on a JASCO FT/IR-4100 Fourier Transform infrared spectrometer. Optical rotation was measured on a JASCO P-1020 polarimeter. Column chromatography was performed with silica gel (200-300 mesh, Qingdao Haiyang Chemical Co., Qingdao, P.R. China), RP-18 reversed-phase silica gel (YMC), and Sephadex LH-20 (Pharmacia). TLC was carried out with precoated silica gel plates (GF-254, Qingdao Haiyang Chemical Co., Qingdao, P.R. China). All solvents were of analytical grade.

### Algal Material

The brown alga *Dictyopteris divaricata* was collected off the coast of Yantai (lat. 37°31’15”N, long. 121°26’59”E), Shandong Province, P. R. China, in July 2008, and a voucher specimen (MBA0807) has been deposited at the Bio-Resource Laboratory of Yantai Institute of Coastal Zone Research for Sustainable Development, Chinese Academy of Sciences.

### Extraction and Isolation

Dried and powdered alga *D. divaricata* (2 kg) was extracted with the mixture of CHCl_3_ and MeOH (1:1, v/v). The concentrated extract was partitioned between H_2_O and EtOAc. The EtOAc-soluble fraction (90 g) was fractioned by silica gel column chromatography [petroleum ether (PE)/EtOAc gradient] to give ten fractions, I-X. Fraction VII, eluted with PE/EtOAc 2:1, was further purified by Sephadex LH-20 (CHCl_3_/CH_3_OH) and RP-18 (CH_3_OH/H_2_O 3:1) column chromatography and preparative TLC (PE/EtOAc 3:1) to afford *1,4-epoxymuurolan-5β-ol* (**1,** 9.1 mg) as a colorless oil; [*α*]^25^_D_ –29.2° (c=0.33, CHCl_3_); IR (KBr) cm^-1^: 3,452, 2,962, 2,954, 2,870, 1,458, 1,377, 1,065; ^1^H-NMR and ^13^C-NMR: see Table 1; ESIMS *m*/*z*: 261 [M+Na]^+^; HRESIMS *m*/*z*: 261.1829 [M+Na]^+^, calcd. for C_15_H_26_O_2_Na, 261.1830. 

### Brine Shrimp Assays

Brine shrimp assays were performed as previously described [10].

## Figures and Tables

**Table 1:** ^1^H and ^13^C-NMR data and ^1^H-^1^H COSY correlations of compound **1** (in CDCl_3_, *δ* in ppm, *J* in Hz).

*No.*	*δ*_C_	*δ*_H_	^1^H-^1^H COSY
1	87.1 s		
2a	34.6 t	1.42 (ddd, 12.5, 9.6, 5.8)	H-2b, H-3a, H-3b
2b		1.94 (ddd, 12.5, 12.1, 4.0)	H-2a, H-3a, H-3b
3a	29.4 t	1.30 (ddd, 12.1, 11.9, 5.8)	H-3b, H-2a, H-2b
3b		2.21 (ddd, 11.9, 9.6, 4.0)	H-3a, H-2a, H-2b
4	85.7 s		
5	85.5 d	3.46 (br s)	H-6
6	56.0 d	1.26 (d, 11.6)	H-5, H-7
7	47.3 d	1.14 (dddd, 12.1, 11.6, 2.1, 1.6)	H-6, H-8a, H-8b
8a	23.7 t	0.89 (dddd, 12.9, 12.6, 12.1, 2.1)	H-7, H-8b, H-9a, H-9b
8b		1.54 (br dd, 12.9, 3.1)	H-7, H-8a, H-9a, H-9b
9a	31.7 t	1.23 (m)	H-8a, H-8b, H-9b, H-10
9b		1.62 (m)	H-8a, H-8b, H-9a, H-10
10	34.9 d	1.59 (m)	H-9a, H-9b, H-14
11	27.3 d	1.80 (hept d, 6.9, 1.6)	H-7, H-12, H-13
12	16.0 q	0.80 (d, 6.9)	H-11
13	21.8 q	0.94 (d, 6.9)	H-11
14	15.3 q	1.01 (d, 6.5)	H-10
15	19.6 q	1.41 (s)	
